# Development and evaluation of a novel MR‐compatible pelvic end‐to‐end phantom

**DOI:** 10.1002/acm2.12455

**Published:** 2018-11-08

**Authors:** Justine M. Cunningham, Enzo A. Barberi, John Miller, Joshua P. Kim, Carri K. Glide‐Hurst

**Affiliations:** ^1^ Department of Radiation Oncology Henry Ford Health System Detroit MI USA; ^2^ Modus Medical Devices Inc. London ON Canada

**Keywords:** MR‐linac, multimodality, physical phantoms

## Abstract

MR‐only treatment planning and MR‐IGRT leverage MRI's powerful soft tissue contrast for high‐precision radiation therapy. However, anthropomorphic MR‐compatible phantoms are currently limited. This work describes the development and evaluation of a custom‐designed, modular, pelvic end‐to‐end (PETE) MR‐compatible phantom to benchmark MR‐only and MR‐IGRT workflows. For construction considerations, subject data were assessed for phantom/skeletal geometry and internal organ kinematics to simulate average male pelvis anatomy. Various materials for the bone, bladder, and rectum were evaluated for utility within the phantom. Once constructed, PETE underwent CT‐SIM, MR‐Linac, and MR‐SIM imaging to qualitatively assess organ visibility. Scans were acquired with various bladder and rectal volumes to assess component interactions, filling capabilities, and filling reproducibility via volume and centroid differences. PETE simulates average male pelvis anatomy and comprises an acrylic body oval (height/width = 23.0/38.1 cm) and a cast‐mold urethane skeleton, with silicone balloons simulating bladder and rectum, a silicone sponge prostate, and hydrophilic poly(vinyl alcohol) foam to simulate fat/tissue separation between organs. Access ports enable retrofitting the phantom with other inserts including point/film‐based dosimetry options. Acceptable contrast was achievable in CT‐SIM and MR‐Linac images. However, the bladder was challenging to distinguish from background in CT‐SIM. The desired contrast for T1‐weighted and T2‐weighted MR‐SIM (dark and bright bladders, respectively) was achieved. Rectum and bone exhibited no MR signal. Inputted volumes differed by <5 and <10 mL from delineated rectum (CT‐SIM) and bladder (MR‐SIM) volumes. Increasing bladder and rectal volumes induced organ displacements and shape variations. Reproduced volumes differed by <4.5 mL, with centroid displacements <1.4 mm. A point dose measurement with an MR‐compatible ion chamber in an MR‐Linac was within 1.5% of expected. A novel, modular phantom was developed with suitable materials and properties that accurately and reproducibly simulate status changes with multiple dosimetry options. Future work includes integrating more realistic organ models to further expand phantom options.

## 1 INTRODUCTION

Radiation therapy (RT) treatments are traditionally planned using computed‐tomography simulation (CT‐SIM) images, with interfraction patient setup verification performed using x‐ray‐based on‐board (OB) imaging techniques. Although CT offers strengths such as providing a direct measurement of electron density for dose calculation and geometric image accuracy, it lacks the excellent soft tissue contrast achievable from magnetic resonance imaging (MRI).^1^ In the male pelvis, MRI has been shown to improve prostate delineation accuracy,^2^, ^3^, ^4^, ^5^ reduce interobserver contouring variability,^3^, ^4^, ^5^, ^6^ improve the localization of the prostate apex,^2^, ^3^, ^4^, ^5^, ^6^ and increase differentiation of the seminal vesicles from the base of the prostate.^2^, ^4^, ^6^ Consequently, treatment planning using MR/CT coregistered images, where delineated soft tissue structures on MR images are transferred onto fused CT images, is often utilized.^2^, ^6^ However, uncertainties on the order of 2 mm are introduced due to this coregistration in the pelvis^7^, ^8^ that may be reduced with MR‐only radiation treatment planning.^9^ MR‐only treatment planning eliminates this coregistration uncertainty while streamlining clinical efficiency.

Furthermore, the implementation of on‐board MR image‐guided radiation therapy (IGRT) systems allows for daily image guidance and real‐time imaging throughout a treatment fraction, which is ideal for managing and monitoring both interfraction and intrafraction motion, respectively, without additional radiation exposure.^10^, ^11^ It has been shown that critical structures and targets within the pelvis are better visualized on MR‐IGRT systems than OB‐CT,^10^ allowing for superior target localization. This, in turn, may lead to improved accuracy of MR‐to‐MR registration and facilitate dose escalation while also offering potential to reduce treatment margins and toxicity to organs at risk.^11^

However, MRI and MR simulation (MR‐SIM) acquisitions typically require longer scanning times than CT, which may result in additional errors being introduced because of anatomical or patient movement.^12^ In the pelvis, multiple uncertainties may arise as a result of patient motion, changes in anatomical structure (position/deformation) due to intrasession bladder filling, and the introduction of patient‐specific distortions due to air in the rectum.^13^ Because of this transient nature, it is currently difficult to characterize the geometric and dosimetric uncertainties that may arise in these new workflows. MR‐compatible phantoms are currently limited for benchmarking these new workflows. Recently, an anthropomorphic multimodality prostate phantom was developed to compare MR‐SIM to CT‐SIM.^14^ The phantom was custom designed with organs (prostate, rectum, bladder, and femoral heads) that adequately generated signal in MR for end‐to‐end testing. However, it was unable to simulate organ filling. Niebuhr et al. performed a thorough material evaluation to create a deformable male pelvic phantom to study adaptive treatment scenarios in MR‐IGRT^15^ with the ability to induce volume variations in both the bladder and rectum. However, sodium salt‐loaded gels used to mimic soft tissue attenuation properties induced severe MR artifacts. In general, these phantoms met their individual design goals but lacked the ability to perform dosimetric verification as required to benchmark MR‐IGRT or MR‐only workflows.

This work describes the development of a novel pelvic end‐to‐end (PETE) MR‐compatible phantom that meets the design goals of benchmarking both MR‐only treatment planning and MR‐IGRT workflows. The phantom is anthropomorphic and modular and enables dosimetric validation. Furthermore, the phantom can simulate different physiological status conditions and may be used to quantify the uncertainties introduced in both MR‐only and MR‐IGRT workflows.

## 2 MATERIALS AND METHODS

### 2.A Phantom geometry

Phantom outer dimensions were determined via retrospective evaluation of axial MR‐SIM T2‐weighted (T2W) Turbo‐Spin‐Echo (TSE) data for 19 prostate cancer patients under an IRB‐approved study. Patients were positioned supine and head‐first, aligned using external LAP lasers (LAP GmbH Laser Applications, Lüneberg, Germany) to right central axis (CAX), left CAX, and anterior CAX tattoos. They were immobilized with their hands placed on chest, feet banded, and knees immobilized in a black leg sponge. The phantom habitus was determined by evaluating data taken in treatment position at the marked isocenter across the cohort. Measurements of the pelvis width, pelvis height, and sacrum external spacing (distance between the posterior edge of the sacrum and the exterior body surface) were taken using the distance measurement tool in the Eclipse Treatment Planning System (Varian Medical Systems, Palo Alto, CA). Because of the high visibility of the pelvic bones in the CT‐SIM dataset, the pelvic skeleton dimensions across the same 19 subjects were also determined using the treatment planning CT for each patient. Measurements for the iliac crest width, femoral head width, greater trochanter width, pelvic skeleton depth, and pelvic skeleton height were also obtained using the distance measurement tool in Eclipse.

### 2.B Internal organ kinematics

**Figure 1:**
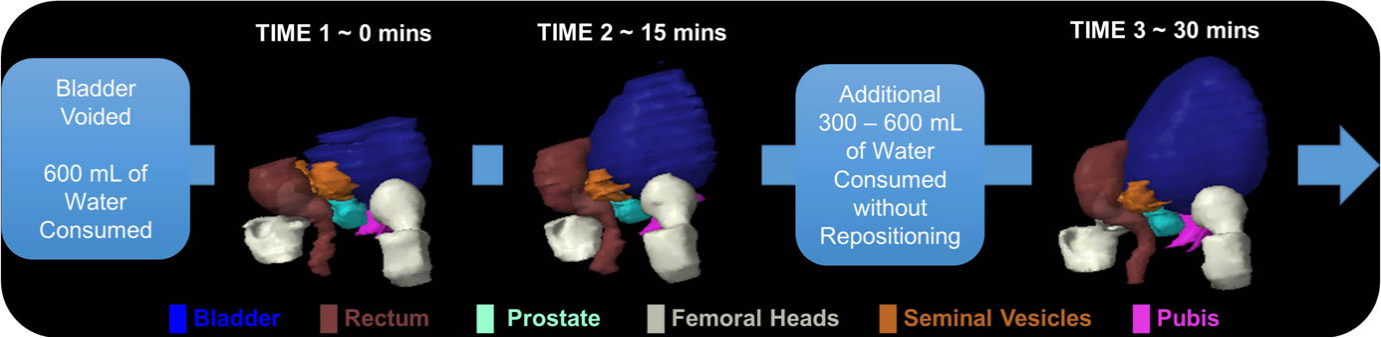
Bladder filling protocol: the patient originally voided their bladder then consumed ~600 mL of water. A T2W MR‐SIM scan was acquired immediately after drinking and 15 min later. After which, the subject consumed an additional 300–600 mL of water and was imaged again a total of 30 min after the initial bladder void. A 3D modeling is shown for each time point, where a much larger longitudinal than lateral growth of the bladder is observed.

**Figure 2:**
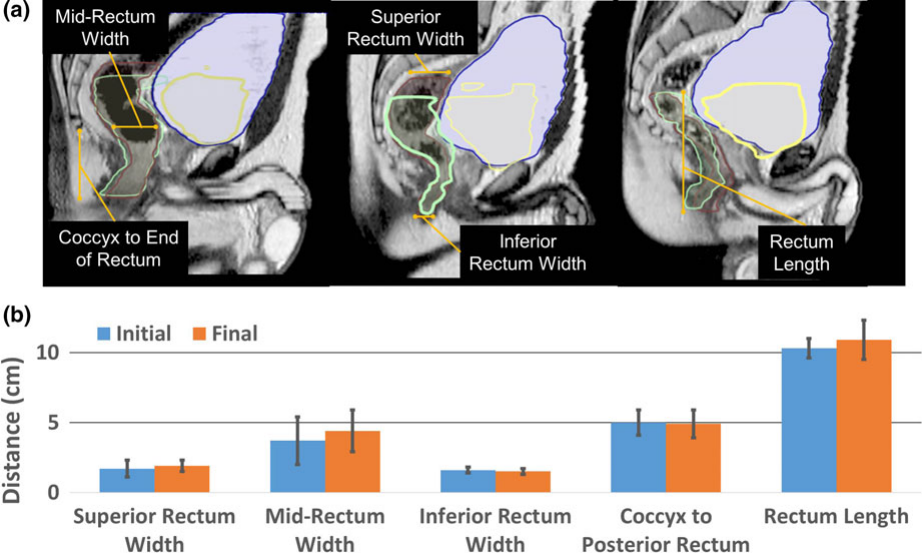
Rectum dimension measurements: (a) visually demonstrates the growth and positioning of the bladder throughout the bladder filling protocol. The initial and final bladder volumes are contoured in blue and yellow, respectively, while the initial and final rectums are contoured in green and brown, respectively. The corresponding initial and final rectum measurements demonstrated in (a) are shown in (b), where a larger rectum length was observed than width.

To quantify the impact of systematic bladder filling on organ volume, location, and displacement, 10 immobilized healthy volunteers underwent a ~45‐min MR‐SIM imaging session using the bladder filling protocol outlined in Fig. 1. Subjects voided their bladder prior to consuming 600 mL of water, T2W sequences were acquired immediately with empty bladders and ~15 min postconsumption with partially full bladders. An additional 300–600 mL of water were consumed with no subject repositioning, and one to two more time points were acquired with full bladders. A single physician delineated the bladder and rectum at each time point following RTOG 0815 criteria.^16^ Temporal datasets were evaluated for the center of mass, shape, and volume of the rectum and bladder with varied filling conditions. To characterize the rectum shape, measurements were obtained as shown in Fig. 2(a) for the width of the anterior, posterior, and middle of the rectum, the length, and the distance from the coccyx to the posterior of the rectum.

### 2.C Phantom materials

#### 2.C.1 Bladder considerations

Two medical balloon catheter assemblies, a 600‐mL maximum internal volume polyisoprene balloon (#CBL7P, Mui Scientific) and a 300‐mL maximum internal volume silicone balloon (#BM‐300‐2, Mui Scientific) were evaluated for preliminary material evaluation for simulating bladder status changes. The polyisoprene balloon was filled with distilled water using a 20‐mL syringe to increasing volumes up to 350 mL while the silicone balloon was filled to 250 mL and imaged in a 1.0‐T Panorama High‐Field Open (HFO) system (Philips Medical Systems, Best, the Netherlands). The general shape, long‐term stability, and filling capacity of each of these balloon assemblies were assessed. To evaluate the potential of filling the silicone balloon to >300 mL, the filling integrity was assessed by filling it repeatedly to 500 mL and visually inspecting the balloon for mechanical/physical changes.

#### 2.C.2 Bone considerations

Two candidate custom pelvic skeletons produced by Stratasys (Stratasys Ltd., Eden Prairie, MN) were custom cast in WC‐788 (clear) and PT8957 (blue) urethane material using average male pelvic geometry, with densities of 1.10 and 1.25 g/mL, respectively. Both pelvises underwent imaging in a Brilliance Big Bore (Philips Health Care, Cleveland, OH) CT‐SIM and a Philips 1.0‐T High‐Field Open (HFO) MR‐SIM. Because cortical bone exhibits short T2 and T2* properties, it is typically undetectable in MRI. However, improved visibility is achieved through the utilization of ultra‐short echo time (UTE) sequences.^17^ Therefore, both pelvises were imaged using a UTE‐Dixon sequence (repetition time (TR)/echo time (TE)/flip angle (*α*) = 11.5/(0.14/3.45/6.9) ms/25°, voxel size = 0.96 × 0.96 × 1.3 mm^3^, and bandwidth = 994 Hz/pixel) to determine if any measurable signal was detected.

### 2.D Phantom evaluation

#### 2.D.1 Scan acquisition

The final phantom build was evaluated across three platforms. CT images were acquired using a Philips Brilliance Big Bore CT‐SIM with the following settings: 120 kVp, 244 mAs, 512 × 512 mm^2^ field of view (FOV), 0.98 × 0.98 mm^2^ resolution, and 2.0‐mm slice thickness. To ensure consistent positioning within each imaging modality, the phantom was aligned via external LAP lasers to the external markings (anterior CAX, left CAX, and right CAX) made during CT‐SIM. MR images were acquired on the MRIdian Linac (ViewRay Inc., Oakwood Village, OH) using a true fast imaging and steady precession (TrueFISP) sequence. This is a fully refocused (refocusing occurs in all three axes) steady‐state sequence with shorter acquisition time, high contrast‐to‐noise, and signal‐to‐noise ratios.^18^ Two 12‐element phased array coils were used for imaging the phantom. A 173‐s scan, with a 45 × 30 × 36 cm^3^ FOV, and 0.15 × 0.15 cm^2^ resolution was acquired.

The MR‐SIM images were acquired using the 1.0‐T MR‐SIM equipped with a flat tabletop using a large, rigid eight‐element phased array coil. Three sequences were acquired: an axial three‐dimensional (3D) T1‐weighted (T1W) fast field echo sequence (TR/TE/*α *= 17.7/6.9 ms/25°, voxel size = 0.96 × 0.96 × 2.5 mm^3^, and bandwidth = 145 Hz/pixel), an axial 3D T2W TSE sequence (TR/TE/*α *= 6591.4/80 ms/90°, voxel size = 0.92 × 0.92 × 2.5 mm^3^, and bandwidth = 202 Hz/pixel), and a sagittal T2W (TR/TE/*α *= 2000/90 ms/90°, voxel size = 0.89 × 0.89 × 3.0 mm^3^, and bandwidth = 202 Hz/pixel).

#### 2.D.2 Simulating bladder and rectal status changes

Across all three imaging platforms, scans were acquired with a constant bladder volume of 250 mL and varying inputted rectal volumes (30, 60, 90, 120, and 150 mL). In the MR‐SIM, additional scans were acquired with a constant rectal volume of 60 mL and varying bladder volumes (90, 150, 250, and 350 mL) ranging from a mostly empty bladder to a mostly full bladder. The fixed rectal and bladder volumes of 60 and 250 mL, respectively, were selected as prostate cancer treatments that are ideally simulated/delivered with a mostly empty rectum and mostly full bladder.^19^ Organ visibility was qualititatively evaluated for each imaging modality. Rectum and bladder volumes were delineated on CT‐SIM and MR‐SIM T2W datasets in MIM Maestro (MIM Software. Inc, Beachwood, OH) to analyze filling accuracy, defined as the difference between expected and measured volumes.

#### 2.D.3 Organ interactions

Contours were generated for the bladder, rectum, and prostate on all MR‐SIM T2W datasets in MIM. Centroid displacements due to volume changes were assessed for each contoured organ. Bladder and rectum diameters [left–right (L‐R) and anterior–posterior (A‐P)] were measured with increased bladder and rectum filling to assess shape changes due to differing filling conditions. Associations between bladder and rectum volumes and resulting centroid displacements were assessed via linear regression.

#### 2.D.4 Reproducibility tests

To assess the reproducibility of organ filling, repeated measures were conducted for rectal volumes of 30 and 60 mL for the CT‐SIM and MR‐linac with a fixed bladder volume of 250 mL. Repeated measures of rectal and bladder volumes of 30 and 250 mL, respectively, were obtained in the MR‐SIM. Reproducibility was assessed by contouring organs in MIM and analyzing the centroid and volume differences over repeated trials.

### 2.E Treatment plan and dosimetry verification

A CT‐SIM was performed with bladder and rectum volumes of 250 and 90 mL, respectively. Isocenter was set to the center of the silicone sponge prostate. The bladder, rectum, and prostate, which were set as the gross tumor volume (GTV), were manually contoured in the ViewRay treatment planning system (TPS) (ViewRay Inc., Oakwood Village, OH). To ensure the chamber location was not in a high‐dose gradient region, a 2.5‐cm margin in the posterior direction, and 1.0‐cm margin in all other directions were added to the GTV to generate a pseudo‐planning target volume (PTV). An 11‐field IMRT 6X Flattening Filter Free (FFF) MR‐Linac treatment plan was generated on the CT dataset using a Monte Carlo dose calculation algorithm based on VMC++^8^ in the ViewRay TPS. Fifty step‐and‐shoot segments were used for a total MU of 756.5 and a target dose constraint of 78 Gy to 95.00% of the PTV, delivered in 39 × 2 Gy fractions. After CT‐SIM, the phantom was setup to scribes on the MR‐Linac, localized with a 173 s, 45 × 30 × 36 cm^3^ FOV, and 0.15 × 0.15 cm^2^ resolution TrueFISP MRI sequence. Couch corrections determined during localization were applied. A point dose measurement was acquired between the prostate and rectum using an MR‐compatible A12 Exradin ion chamber (Standard Imaging Inc., Middleton, WI) and compared to the TPS.

## 3 RESULTS

### 3.A Phantom geometry

Table 1 best summarizes the average dimensions of the male pelvis habitus and pelvic skeleton geometry measured from the 19‐patient cohort. The final male pelvis phantom external acrylic casing was 38.1 cm wide (4.3% less than expected) and 23.0 cm tall. The final phantom skeletal structure greater trochanter width had the largest percent difference (11.2%) from the desired values. However, all skeletal dimensions were within the range of measurements taken from the patient population data.

**Table 1:** Patient population and final phantom pelvic habitus and skeleton dimensions

Measurement	Patient population dimensions (cm) mean ± SD (range)	Final phantom dimension (cm)	Percent difference
Body width (left–right)	36.5 ± 1.9 (33.6–40.0)	38.1	4.3
Body height (anterior–posterior)	23.0 ± 1.4 (21.2–25.6)	23.0	0
Sacrum—external Spacing	1.6 ± 0.4 (1.0–2.2)	1.6	0
Iliac crest width	25.5 ± 2.1 (22.8–28.8)	27.2	6.4
Femoral head width	22.2 ± 1.3 (20.0–25.5)	23.5	5.7
Greater trochanter width	30.2 ± 1.6 (26.9–34.4)	33.8	11.2
Pelvic depth	14.7 ± 1.0 (12.8–17.0)	14.2	3.5
Pelvic height	20.9 ± 1.4 (17.4–23.4)	20.8	0.5

Values are given as a mean of the prostate patient population (n = 19).

SD, standard deviation.

Percent difference calculated between the mean of the patient population dimensions and final phantom dimensions.

### 3.B Internal organ kinematics

To quantify internal status changes, bladder filling data from 10 human subjects yielded an average bladder volume difference of 87% between empty (81.9 ± 66.9 mL) and full (383.0 ± 346.7 mL) bladders. Figure 1 shows a 3D rendering of the volumetric bladder and rectum at each time point for a representative case, highlighting the large longitudinal displacement between initial and final bladder volumes. Bladder centroids translated 13.2 ± 11.7 mm superiorly, 1.7 ± 7.5 mm anteriorly, and 1.4 ± 4.2 mm laterally (left) over the cohort. On average, rectum centroids moved 1.7 ± 10.7 mm superiorly, 1.2 ± 2.8 mm posteriorly, and 0.2 ± 1.0 mm laterally (left). Figure 2(a) demonstrates the position and growth of the bladder and rectum over time for three of the subjects who participated in the bladder filling study. Rectal volumes ranged between 30 and 270 mL (105 ± 65 mL). The results for the width and length of the rectum contour as well as the distance from the coccyx to the posterior of the rectum are shown in Fig. 2(b). The rectum yielded the largest increase in width in its center between the initial and final time points, demonstrating that a balloon with a larger length than width, and with a wide central axis was needed to simulate the rectum. A coccyx to posterior rectum distance of approximately 5 cm was recommended for rectal balloon placement within the phantom.

### 3.C Phantom materials

#### 3.C.1 Bladder considerations

The silicone and polyisoprene balloons were filled to a maximum volume of 250 and 350 mL of distilled water, respectively, without bursting. After one filling cycle, the polyisoprene balloon showed slight deterioration and mechanical changes. The silicone balloon was more robust during filling experiments, suggesting that it would be good for long‐term use within the phantom. The silicone balloon was filled to >380 mL with little to no air bubbles present. Therefore, the silicone balloon was chosen for use within the phantom as it addressed the concern of deterioration of polyisoprene over time with exposure to water and oxygen and was able to achieve the desired volumes. Additionally, silicone, in terms of electron density and MR signal is a suitable substitute material for exterior organ shells.^15^ The barbed fitting used to tether the balloon to the catheter at each end withstood balloon pressure at maximum volume.

#### 3.C.2 Bone considerations

Both mold‐cast pelvises had anthropomorphic shapes, but the clear urethane pelvis structure did not include femoral heads and was >12% different than desired in pelvic width and depth. The clear pelvis also had a CT number of 60 HU, which is considerably less than the CT number of cancellous bone (262 HU).^20^ The blue dyed urethane pelvis included femoral heads, was appropriately sized, and exhibited a CT number closer to that of cancellous bone (213 HU). Neither pelvis generated a signal in MRI sequences, particularly in the UTE acquisition, and neither approximated cortical bone (1454 HU).^20^ Nevertheless, the blue urethane was selected for the initial phantom build due to its higher CT number.

### 3.D Phantom build

**Figure 3:**
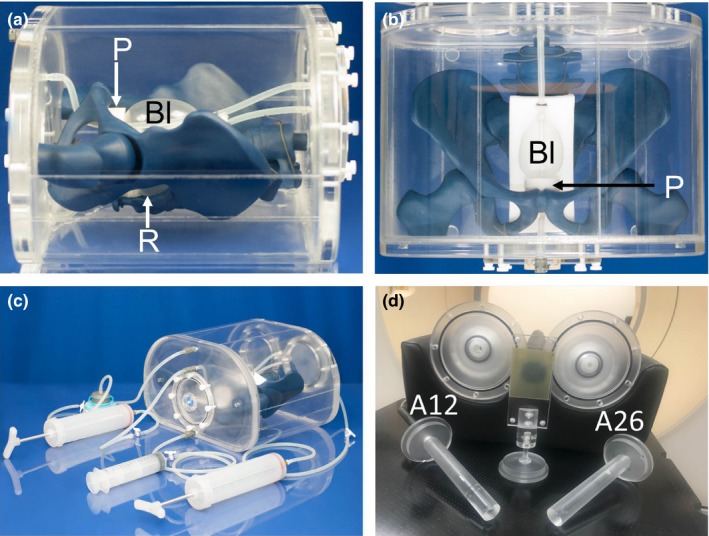
Final phantom build: (a) Sagittal and (b) coronal view, where the bladder (Bl) and rectum (R) are represented by the chosen silicone balloon and the prostate (P) by a cylindrical silicone sponge. (c) Four devices (2 × 400 mL syringe, 140‐mL syringe, and 1000‐mL flexible reservoir) used to induce bladder and rectal status changes. (d) Two replaceable endcaps that allow for the A12 and A26 Exradin MR‐compatible ion chambers to be inserted into the phantom and their corresponding inserts, as well as the film cassette.

The final phantom build is shown Figs. 3(a)–3(c), with average male pelvic anatomy (height/width = 23.0/38.1 cm). Final material candidates were as follows: 300‐mL silicone balloons for the bladder (#BM‐300‐2), and rectum (#BM‐300‐3), and blue dyed urethane (PT8957) for the pelvic structure. Each balloon was tethered at both ends (axially) by their catheter to the pelvic structure by thin silicone rubber chord to prevent them from floating freely throughout the phantom. The bladder and phantom habitus were filled with distilled water doped with 7 and 15 mg/L concentrations of Mn^2+^ (as MnCl_2_:4H_2_O) to achieve a relaxation time of ~900 ms (urine) and ~300 ms (fat/muscle), respectively. Figure 3(c) highlights the bladder and rectal filling assembly consisting of two 400‐mL syringes used to induce bladder volume changes, a 140‐mL syringe used to fill the rectum with air, and a 1000‐mL flexible reservoir (Hydrapak, Oakland, CA) that can be easily removed via quick connect/disconnect fittings. The expansion device filled with the same concentration [15 mg/L of Mn^2+^ (as MnCl_2_:4H_2_O))] of distilled water as the phantom habitus was used to account for water displacement caused by varying internal volumes, to decrease internal pressure on the phantom casing, and also to reduce the amount of air bubbles.

A quarter‐inch‐thick polyvinyl alcohol (PVA) open cell sponge was inserted between the bladder and rectum to systematically tether them together and mimic connective tissue. A silicone sponge was formed into a cylindrical shape to represent the prostate with a central hole for the 4‐mm‐inner diameter and 7‐mm‐outer diameter silicone rubber tubing bladder catheter (urethra). Three ~10‐cm‐diameter removable end caps were added to the exterior of the phantom to enable access for modular changes such as inserting dosimetry equipment and substituting organs while the skeletal structure is rigidly affixed. Figure 3(d) highlights interchangeable end caps designed to fit MR‐compatible A12 and A26 Exradin ion chambers. Additional dosimetry inserts to accommodate MR‐compatible chambers and a 2″ × 2.5″ film cassette can also be utilized.

### 3.E Phantom evaluation

#### 3.E.1 Simulating bladder and rectal status changes

**Figure 4:**
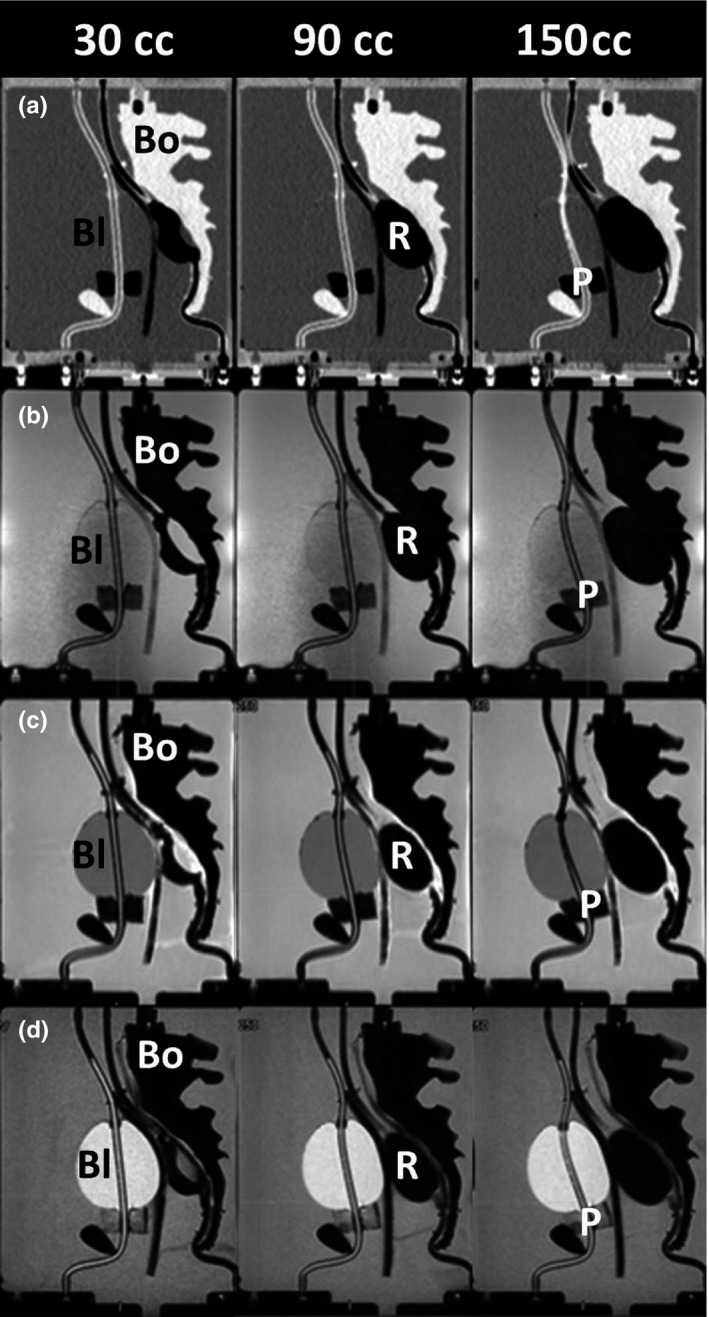
Increasing rectal volumes: sagittal view for increasing rectum volumes (30, 90, and 150 mL) for (a) CT‐SIM, (b) MR‐Linac, (c) T1W MR‐SIM, and (d) T2W MR‐SIM. The bladder (Bl), rectum (R), prostate (P), and bone (Bo) are labeled in each modality.

Sagittal views of the CT‐SIM datasets at inputted rectal volumes (30, 90, and 150 mL) are shown in Fig. 4(a). The rectum, urethra, bone, and phantom filling exhibited contrasts as expected for these tissue types. However, the bladder was almost indistinguishable from surrounding material and was very difficult to visualize in the CT‐SIM images. Delineated rectal volumes differed by a maximum of 14% (<5 mL) from the expected rectal volume of 30 mL. Overall, a difference of 3.3 ± 7.0% (0.6 ± 3.8 mL) was obtained over all rectal volumes with excellent agreement between expected volumes and delineated volumes.

Figure 4(b) shows the sagittal TrueFISP MR‐Linac images for increasing rectal volumes. In this modality, the bladder was slightly darker than background and easier to distinguish. The open cell silicone sponge prostate changed proton density to give MR contrast and was visible just beneath the bladder. As expected, the rectal air and pelvic bones generated no MR signal, rendering the organ boundaries indistinguishable from each other. As a result, a quarter‐inch‐thick PVA sponge was inserted between the pelvis and rectum to introduce a barrier and differing contrast for future imaging studies. This modification was possible due to the modular design of the phantom allowing the phantom interior to be retrofitted when needed.

**Figure 5:**
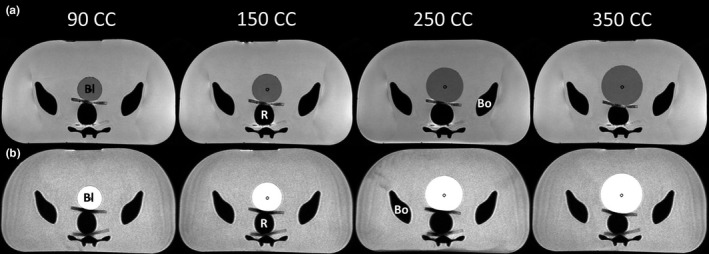
Increasing bladder volumes: axial view for increasing bladder volumes (90, 150, 250, and 350 mL) for (a) T1W MR‐SIM and (b) T2W MR‐SIM. The bladder (Bl), rectum (R), and bone (Bo) are labeled.

The T1W and T2W MR‐SIM images are shown in Figs. 4(c) and 4(d) for rectal volumes of 30, 90, and 150 mL. The PVA sponge successfully generated a barrier to differentiate between the rectum and bones in the T1W and T2W images. The expected T1W contrast (darker bladder, brighter background) and T2W contrast (brighter bladder, darker background) were observed in the MR‐SIM sequences. Axial views of the T1W and T2W datasets for increasing bladder volumes (90, 150, 250, and 350 mL) are shown in Figs. 5(a) and 5(b). Delineated bladder volumes differed by a maximum of 11% (10 mL) from the expected bladder volume of 90 mL. Across all bladder volumes, an overall difference of 3.1 ± 5.6% (2.5 ± 6.4 mL) was obtained with excellent agreement between expected and measured.

#### 3.E.2 Organ interactions

**Figure 6:**
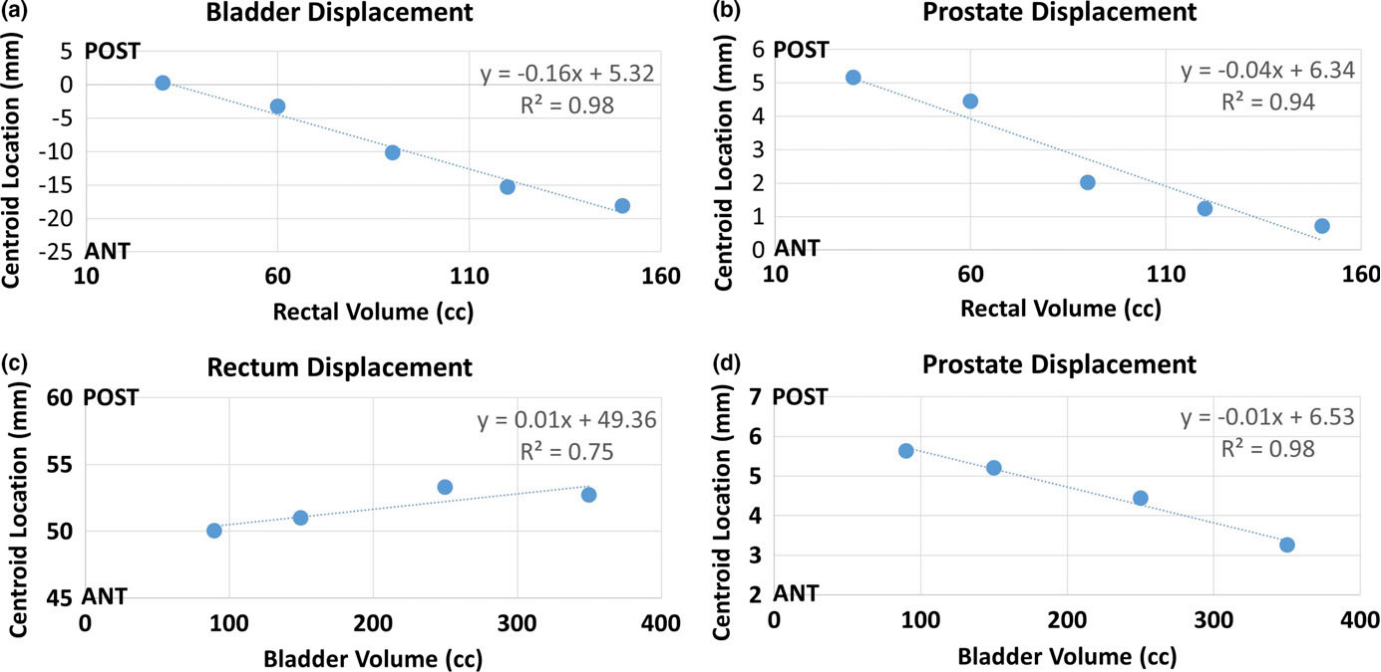
Centroid displacements: (a) bladder and (b) prostate anterior–posterior centroid displacement with increasing rectal volume. (c) Rectum and (d) prostate anterior–posterior centroid displacement with increasing bladder volumes.

With increasing rectal volumes, both the bladder‐ and prostate‐simulated organs translated in the anterior direction as highlighted by the MR‐Linac and MR‐SIM sagittal images in Figs. 4(b)–4(d). Negligible centroid shifts (<2.5 mm) were observed in the superior–inferior (S‐I) and L‐R directions. The scatter plot shown in Figs. 6(a) and 6(b) summarizes the A‐P bladder and prostate centroid displacements for increasing rectal volumes. The bladder and prostate translated 18.5 and 4.5 mm toward the anterior of the phantom between initial (30 mL) and final (150 mL) rectal volumes, respectively. Strong, negative associations (R^2^ = 0.94–0.98, *P* < 0.01) were observed between increasing rectal volumes and bladder A‐P and prostate A‐P displacement. Between the initial (30 mL) and final (150 mL) rectal volumes, a 6‐mm reduction in bladder width (A‐P) at the centroid location was observed due to the compression induced on the bladder with increased rectal volume.

Figures 6(c) and 6(d) best summarize the A‐P centroid displacements for the rectum and prostate in response to bladder filling. The prostate translated 2.4 mm toward the phantom anterior between initial and final bladder volumes (90 and 350 mL, respectively), where a strong, negative correlation (R^2^ = 0.98, *P* = 0.0075) was observed between prostate displacement (A‐P) and increasing bladder volumes. Negligible prostate centroid displacements (<1.5 mm) were observed in the S‐I and L‐R directions with increased bladder size. With increased bladder volumes, minimal rectal displacements were observed in the A‐P direction (<3 mm), whereas negligible rectum centroid displacements were observed in the S‐I and L‐R directions (<1.5 mm). However, Fig. 5 highlights that increasing the bladder volume from 90 to 350 mL resulted in a corresponding rectal compression in the A‐P direction. A 9‐mm decrease (A‐P) and 6‐mm increase (L‐R) in the rectum width was observed.

#### 3.E.3 Reproducibility tests

**Figure 7:**
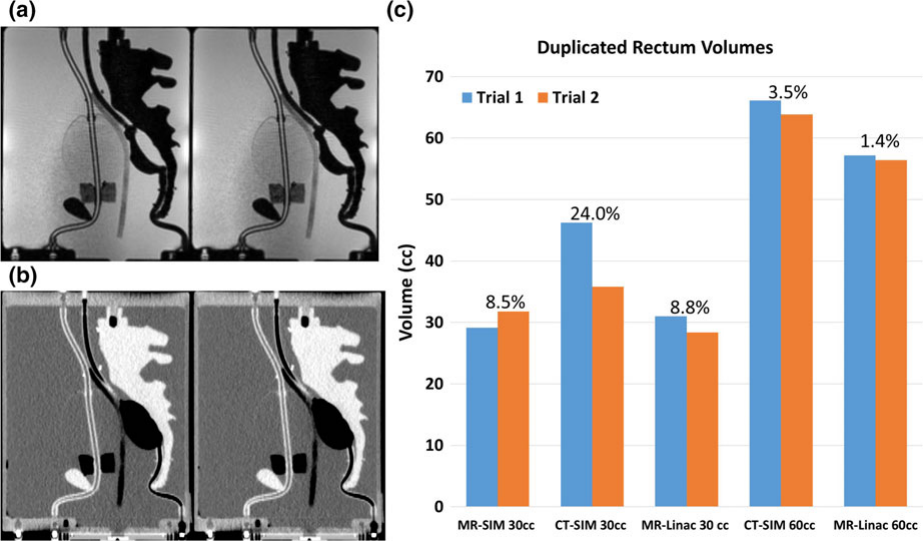
Reproduced sagittal volumes: (a) MR‐Linac 30 mL and (b) CT‐SIM 60 mL datasets for reproduced rectum trials. (c) Volume changes for the bladder and rectum from trials 1 and 2 of duplicated rectal volumes in each modality, with the corresponding percent volume changes.

Sagittal views of the reproduced 30‐ and 60‐mL rectal volumes in the MR‐Linac and CT‐SIM are shown in Figs. 7(a) and 7(b), where similar rectal shapes were observed in each duplicated system. Volume changes observed in each reproduced volume trial are shown in Fig. 7(c), where smaller rectal volumes were more challenging to reproduce due to unpredictable balloon contraction/expansion with small volumes. The reproduced 30‐mL (n = 3) and 60‐mL (n = 2) rectum volumes exhibited 1.32 ± 0.49 and 0.36 ± 0.46 mm average vector centroid changes and 5.00 ± 4.14 mL (~13.8%) and 1.53 ± 1.06 mL (~2.4%) volume differences, respectively, between trials. In MR‐SIM, a reproduced bladder volume of 250 mL exhibited a 1.6 mL (~0.6%) volume change and a 0.70‐mm centroid change between trials.

### 3.F Dosimetry verification

**Figure 8:**
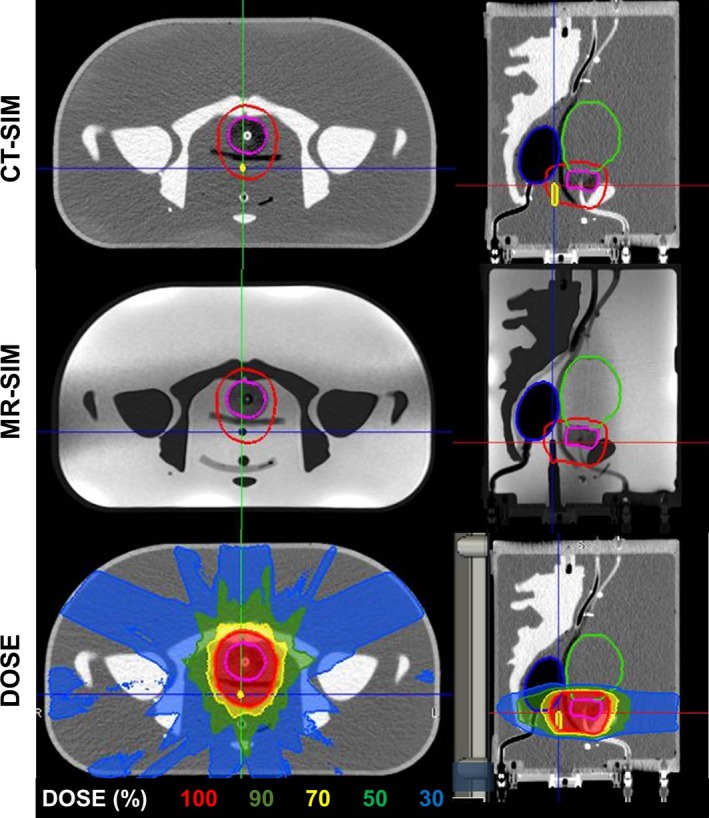
Treatment plan: axial and sagittal views of the CT‐SIM, 0.35 T MR‐SIM, and dose for an 11‐field IMRT 6XFFF MR‐Linac treatment plan at the same slice in the phantom. Contours of the bladder (green), rectum (blue), gross tumor volume (pink), planned target volume (red), and ion chamber (yellow) are shown. The chamber is not delineated in the MR‐SIM to highlight its visibility for localization.

The MR‐SIM, CT‐SIM, and corresponding treatment plan calculated on the CT‐SIM dataset are shown in Fig. 8. The ion chamber was visible and contoured in MR‐SIM images. The PTV was expanded in the posterior direction to ensure the ion chamber did not fall into a high dose gradient region. The plan met all prescription and organ at risk dose constraints. The measured point dose between the rectum and prostate was 212.8 cGy, 1.5% different from the expected 216.0 cGy TPS dose at the chamber location.

## 4 DISCUSSION

With the emergence of MR‐only treatment planning and MR‐IGRT, the need exists to develop an MR‐compatible pelvic phantom that is sophisticated enough to benchmark the uncertainties introduced in these workflows. An anthropomorphic and modular end‐to‐end pelvis phantom was designed and evaluated, with the ability to simulate accurate and reproducible bladder and rectal physiological status conditions. Additionally, the modularity of the phantom permits the ability to perform dosimetric validation using both point and film‐based dosimetry options.

This work introduced several added features to the currently available MR‐compatible pelvic phantoms. Sun et al. incorporated solid polymethylmethacrylate (PMMA) structures to represent the rectum and bladder that did not offer the ability to deform.^14^ As it is more representative of the pelvic region to simulate anatomical position variation due to volume variations in the rectum and bladder, PETE incorporated both a fluid‐fillable bladder and air‐fillable rectum. Previously, Niebuhr et al. and Kadoya et al. incorporated 3D‐printed deformable bladders that accurately represented the organs anatomically, but mechanical wear and tear resulted in structures needing to be replaced frequently.^15^ Additionally, at small volumes, the bladder did not deform well as substantial amounts of air remained in the bladder.^21^ The silicone medical balloons used in PETE to represent the bladder and rectum addressed these concerns, as the bladder performed well at low volumes with little presence of air and did not deteriorate rapidly with continued use. A benefit of this design method is that one can simulate different physiological status changes at low and high volumes and measure potential dosimetric and geometric variations that arise due to anatomical structure position as a result of intrasession bladder and rectal filling. The small volume and centroid changes obtained from the accuracy and reproducibility studies suggest that the robust phantom design allowed for precise and reliable reproduced organ states with little organ displacement at different time points. The phantom was also able to induce organ shape changes and displacements, respectively, with increasing rectal and bladder volumes.

Additionally, previous pelvic phantoms offered limited dosimetry options such as optically stimulated luminescent dosimeters (OSLD). Our phantom was designed to enable dosimetric verification of treatment plans via compatibility with MR‐compatible ion chambers, with a dosimetric point dose agreement to <1.5% from expected for an A12 ion chamber. Due to the modular design and endcaps, the insertion of Gafchromic film for planar dose verification or A26 small ion chamber can be explored in future work. Other future work can include incorporating inserts to include OSLDs or a 3D‐printed hollow shelled deformable prostate surrogate as opposed to the cylindrical silicone prostate. This prostate volume may be filled with either a doped agarose gel^15^ that exhibits similar MR signal generation properties (T1 = 1317 ± 85 ms and T2 = 88 ± 0 ms at 1.5 T)^22^ as the prostate or a gel with dosimetric properties. Gel dosimetry has shown promise for measuring 3D dose distributions^23^ and may prove useful within the prostate casing.

A major obstacle with MR‐compatible phantoms is simulating skeletal anatomy with materials that accurately generate tissue specific signal in MRI. Sun et al.*,* was able to represent the low MR signal intensity of the femoral heads by filling spherical PMMA structures.^14^ However, they did not incorporate a structure to represent the rest of the skeletal anatomy. Niebuhr et al. used a 3D‐printed hollow bone case filled with a combination of Vaseline and K_2_HPO_4_ to accurately represent inner bone.^15^ This method generated the signal intensity of inner and outer bone in MRI but lacked the attenuation properties of outer bone in CT. More recently, Soliman et al. used a human skull in a realistic head phantom, which was advantageous as it depicted the properties of cortical bone in UTE sequences that are difficult to simulate.^24^ The skeletal anatomy in PETE was represented by a blue dyed urethane pelvis that was unable to generate the MRI signal characteristics of human cortical bone. However, Rai et al. has identified a 3D printable solid resin material that has similar signal properties to cortical bone in UTE MRI sequences.^25^ This resin material has been successfully used to replicate skeletal anatomy in other phantoms and may prove useful in future generations of the PETE phantom. Adoption of this resin into a new 3D printed pelvic skeleton structure may improve synthetic CT generation and dose calculation performed in an MR‐only workflow.

Additional limitations exist in the phantom design, including only considering male anatomy for phantom and organ geometry. Male anatomy was simulated as the MR‐only RT is FDA approved or CE marked and in clinical use for the treatment of prostate cancer.^26^, ^27^ While the modular phantom does enable the introduction of 3D printed organs for female anatomy (uterus, cervix, etc.), as other deformable phantoms have,^21^ the current pelvic skeleton geometry does not accurately represent the average female anatomy. The pelvis structure was modeled after the male pelvis, which is taller and narrower than the female pelvis, with a 7% difference in width to height ratios.^28^ Future extensions of this work include incorporating a penile bulb and reorganization of internal organs so that the rectum interacts more closely with the prostate.

Despite some of the above limitations, a novel, anthropomorphic, and modular pelvic phantom with the ability to simulate bladder and rectal status changes was developed and validated. Potential future clinical applications of this phantom include the benchmarking of MR‐to‐MR deformable image registration algorithms, evaluation of MR‐based adaptive workflows, quantifying distortions in MR images due to susceptibility effects at air‐tissue interfaces, and evaluating the electron return effect for MR‐IGRT.

## 5 CONCLUSION

A novel end‐to‐end pelvis phantom has been developed to validate MR‐only and MR‐IGRT workflows, with the ability to perform both dosimetric and geometric evaluations.

## CONFLICTS OF INTEREST

The submitting institution holds research agreements with Philips Healthcare, ViewRay Inc., and Modus Medical Devices.
